# Titanium Hydride Nanoplates Enable 5 wt% of Reversible Hydrogen Storage by Sodium Alanate below 80°C

**DOI:** 10.34133/2021/9819176

**Published:** 2021-12-14

**Authors:** Zhuanghe Ren, Xin Zhang, Hai-Wen Li, Zhenguo Huang, Jianjiang Hu, Mingxia Gao, Hongge Pan, Yongfeng Liu

**Affiliations:** ^1^State Key Laboratory of Silicon Materials and School of Materials Science and Engineering, Zhejiang University, Hangzhou 310027, China; ^2^Hefei General Machinery Research Institute, Hefei 230031, China; ^3^School of Civil & Environmental Engineering, University of Technology Sydney, 81 Broadway, Ultimo, NSW 2007, Australia; ^4^School of Chemistry and Chemical Engineering, Yantai University, Yantai 264005, China; ^5^Institute of Science and Technology for New Energy, Xi'an Technological University, Xi'an 710021, China

## Abstract

Sodium alanate (NaAlH_4_) with 5.6 wt% of hydrogen capacity suffers seriously from the sluggish kinetics for reversible hydrogen storage. Ti-based dopants such as TiCl_4_, TiCl_3_, TiF_3_, and TiO_2_ are prominent in enhancing the dehydrogenation kinetics and hence reducing the operation temperature. The tradeoff, however, is a considerable decrease of the reversible hydrogen capacity, which largely lowers the practical value of NaAlH_4_. Here, we successfully synthesized a new Ti-dopant, i.e., TiH_2_ as nanoplates with ~50 nm in lateral size and ~15 nm in thickness by an ultrasound-driven metathesis reaction between TiCl_4_ and LiH in THF with graphene as supports (denoted as NP-TiH_2_@G). Doping of 7 wt% NP-TiH_2_@G enables a full dehydrogenation of NaAlH_4_ at 80°C and rehydrogenation at 30°C under 100 atm H_2_ with a reversible hydrogen capacity of 5 wt%, superior to all literature results reported so far. This indicates that nanostructured TiH_2_ is much more effective than Ti-dopants in improving the hydrogen storage performance of NaAlH_4_. Our finding not only pushes the practical application of NaAlH_4_ forward greatly but also opens up new opportunities to tailor the kinetics with the minimal capacity loss.

## 1. Introduction

Hydrogen storage, bridging hydrogen generation and hydrogen application, plays a crucial role in a future hydrogen energy society [1–4]. Distinct from the matured technologies of compressed and liquefied hydrogen, solid state hydrides can realize higher hydrogen density under moderate pressures and temperature. Metal complex hydrides have attracted tremendous attention as the most promising hydrogen storage candidates because of their high gravimetric and volumetric hydrogen densities [5–8]. Sodium alanate, NaAlH_4_, is a typical complex hydride possessing 7.4 wt% of hydrogen capacity and favorable thermodynamics [9–12]. However, the sluggish kinetics results in high operation temperature and poor reversibility for hydrogen storage in NaAlH_4_, therefore limiting its practical on-board applications.

**Figure 1:**
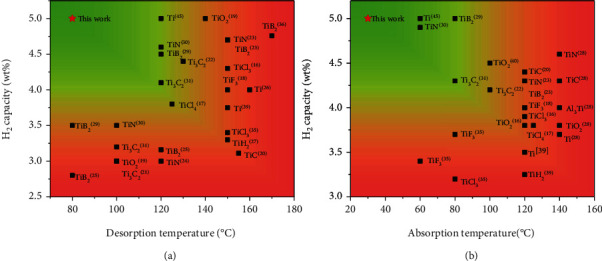
Comparison of hydrogen desorption (a) and absorption (b) performance of NaAlH_4_ doped with various Ti-based catalysts.

Catalyst doping has been proved a feasible approach to help reducing the kinetic barriers of hydrogen storage reactions in metal hydrides. Transition metals and their compounds, especially Ti-based dopants, were found to have the ability to promote fast dissociation and recombination of hydrogen molecules [12–14]. In this respect, Bogdanović and Schwickardi contributed an important breakthrough by introducing a few millimoles of Ti(OBu)_4_ or TiCl_3_ into NaAlH_4_, which enabled reversible hydrogen storage with NaAlH_4_ at moderate conditions [15]. After that, a variety of Ti-based species have been explored and evaluated, including halides, oxides, nitrides, borides, carbides, hydrides, alloys, and elemental metals (Figure 1) [16–31]. In most cases, the Ti-species tends to react with NaAlH_4_ to form Ti*_x_*Al*_y_*, which shows significant catalytic effect on the de-/rehydrogenation. Although most of the Ti-based species exhibit positive effects on the improvement of kinetics, the reduced hydrogen capacity becomes another important issue, especially for the heavy dopants [32]. More importantly, dopants with high valent Ti are readily reduced to the low valence and even to metal state of zero-valence during ball milling with NaAlH_4_, while the anions tend to combine with Na^+^ to form hydrogen inert compounds, consequently further lowering the available hydrogen capacity of the whole composite [16, 33, 34]. As a result, the reversible hydrogen capacity remains only 3-4 wt% for Ti-doped NaAlH_4_ system [35–37]. This is far from 5.6 wt% of theoretical value while NaAlH_4_ decomposes to NaH and Al. Therefore, it is in great need to tackle the abovementioned trade-off issue between reaction temperature and hydrogen capacity of NaAlH_4_-based hydrogen storage materials.

Titanium hydride, TiH_2_, with Ti being already in low valent state and containing hydrogen itself, is expected to be a better candidate of dopant in comparison with other Ti-based compounds. More encouragingly, considerable studies show that the in situ formed TiH_2_ is a catalytic active phase in the Ti-based compound-modified NaAlH_4_ systems [38–43]. For example, Gross et al. observed the conversion of NaH/Al to NaAlH_4_ at 130°C and 82 atm H_2_ with the presence of TiH_2_, indicating a remarkable improvement of hydrogenation properties [38]. Kang et al. reported the in situ generation of TiH_2_ after mechanical milling of metallic Ti powder with NaH/Al mixture under H_2_ atmosphere [39]. A similar phenomenon was also observed during hydrogenation of the TiO_2_-modified NaAlH_4_ system [40]. Moreover, theoretical predications supported the creation of Ti-H bonds via extracting hydrogen atoms from the accessible AlH_4_/AlH_3_ groups [41–43]. However, introduction of commercial TiH_2_ into NaAlH_4_ seemed not very effective (only releasing 3.3 wt% H_2_ within 10 h at 150°C), which may be due to the large TiH_2_ particle, and thus, the catalytic interactions between TiH_2_ and NaAlH_4_ were limited [27]. It is therefore an open question to trigger the high catalytic activity of TiH_2_ that would reduce the reaction temperature and keep a high hydrogen capacity of NaAlH_4_ simultaneously.

In this work, we develop a novel facile sonochemical process for the fabrication of two-dimensional (2D) TiH_2_ nanoplates. Ultrasound was used to drive the formation of nanometer TiH_2_ on graphene by reacting TiCl_4_ with LiH in THF solution, thanks to the high solubility of LiCl. Well-defined TiH_2_ nanoplates with a lateral size of ~50 nm and thickness of ~15 nm on graphene (denoted as NP-TiH_2_@G) were successfully obtained. Outstanding catalytic activity for hydrogen storage reaction of NaAlH_4_ was found to be related to the significantly enhanced surface area and excellent dispersibility in comparison with commercial TiH_2_ in microscale. Full dehydrogenation and rehydrogenation were achieved, respectively, at 80°C and 30°C, with a practical capacity of 5 wt% for NaAlH_4_ doped with 7 wt% NP-TiH_2_@G. To the best of our knowledge, this is the first example that NaAlH_4_ can reversibly store hydrogen in the working temperature range of proton exchange membrane fuel cell (PEMFC) with the highest capacity (Figure 1). Such outstanding hydrogen storage performance of NaAlH_4_ meets the requirement for on-board hydrogen storage application.

## 2. Results

### 2.1. Preparation of TiH_2_ Nanoplates

**Figure 2:**
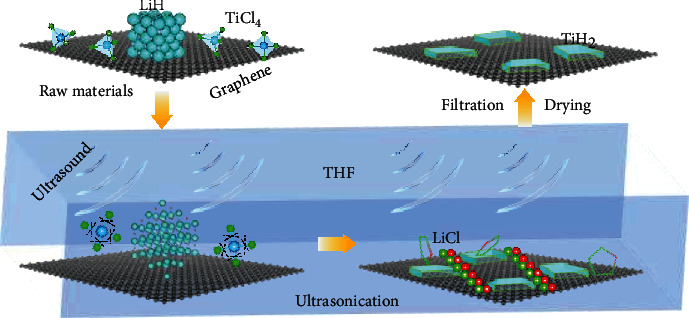
Schematic illustration for the preparation process of TiH_2_ nanoplates.

The process for the preparation of TiH_2_ nanoplates was developed, as illustrated schematically in Figure 2, based on the following chemical reaction.

(1) $$\begin{array}{c} {\text{TiCl}}_{4}+4\text{LiH}\overset{\text{THF}}{\underset{\text{Ultrasonification}}{⟶}}{\text{TiH}}_{2}+4\text{LiCl}+{\text{H}}_{2}↑ \end{array}$$

All sample handling was conducted in an Ar-filled glove box. Firstly, stoichiometric titanium tetrachloride (TiCl_4_) and lithium hydride (LiH) along with a certain amount of graphene were added to tetrahydrofuran (THF) solution. Subsequently, the sonochemical process was conducted for 4 h at 40 kHz with continuous stirring. Finally, the solid-state product was obtained after filtrating, washing, and drying.

**Figure 3:**
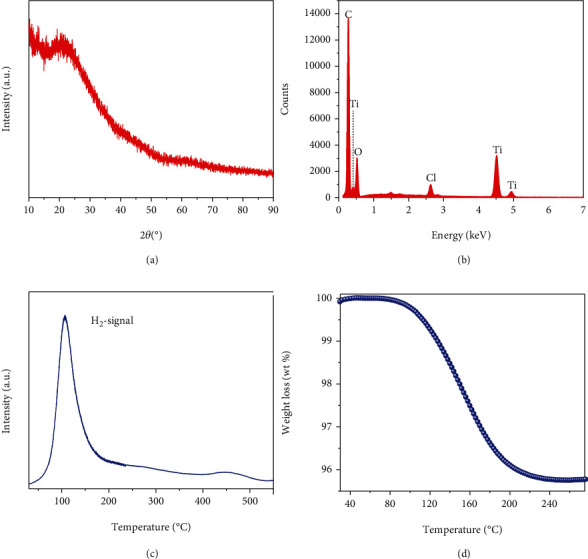
(a) XRD pattern, (b) EDS spectrum, (c) TPD-MS signal, and (d) TGA curve of as-prepared solid products of the sonochemical reaction between TiCl_4_ and LiH in THF.

Only a broad diffraction peak at around 25° with high background was observed in the X-ray diffraction (XRD) profile (Figure 3(a)), suggesting that the solid-state product was in amorphous or nanocrystalline state. Energy dispersive spectroscopy (EDS) analysis revealed that it was mainly composed of Ti and C in addition to a traced amount of Cl and O as impurities (Figure 3(b)). Further Raman characterization indicated that the C signal could reasonably be attributed to graphene from the characteristic D-band and G-band at 1340 and 1590 cm^−1^, respectively (Figure S1, Supporting Information). More importantly, H_2_ emission was detected by mass spectroscopy (MS) while heating the solid-state product (Figure 3(c)). A sample in the absence of graphene was prepared according to reaction (1) with the same process to determine exactly the H content. Thermogravimetric analysis (TGA) results indicated around 4 wt% of weight loss (Figure 3(d)), agreeing well with the H content in TiH_2_. It is worth noting that the peak temperature for the hydrogen release of the solid product is around 120°C (Figure 3(c)), much lower than that of TiH_2_ in microscale (>500°C) [44]. Suggesting the successful synthesis of nanosized TiH_2_. Furthermore, the generation of H_2_ as a gaseous product (Figure S2, Supporting Information) and the formation of LiCl in the filtrate (Figure S3, Supporting Information) as byproducts of reaction (1) were confirmed in the sonochemical process. Thus, the resultant solid-state product consisted of nanosized TiH_2_ and graphene.

**Figure 4:**
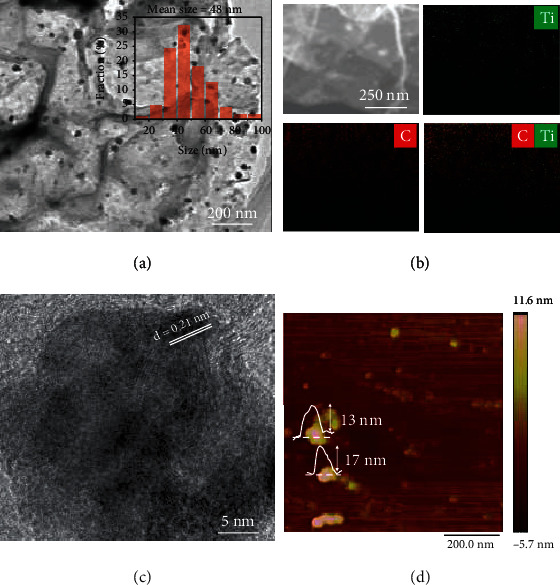
(a) TEM image, (b) EDS mapping, (c) HRTEM image, and (d) AFM image of NP-TiH_2_@G. Inset on (a) is the corresponding particle size distribution.

Figure 4 shows the morphology of the prepared TiH_2_. As shown in Figure 4(a), a large number of black nanoplates with ~50 nm of average size dispersed on graphene can be observed from the transmission electron microscope (TEM) image. EDS mapping indicated that these nanoplates were mainly composed of Ti and C elements (Figure 4(b)). High-resolution TEM (HRTEM) clearly presents the fringes of interplanar spacing of 0.21 nm (Figure 4(c)), which corresponds to the (002) planes of TiH_2_. The TEM observations, therefore, strongly prove the successful synthesis of graphene-supported TiH_2_ nanoplates (denoted as NP-TiH_2_@G hereinafter) by a newly developed sonochemical process. The thickness of the prepared TiH_2_ nanoplates was determined as ~15 nm by atomic force microscope (AFM) measurement (Figure 4(d)).

**Figure 5:**
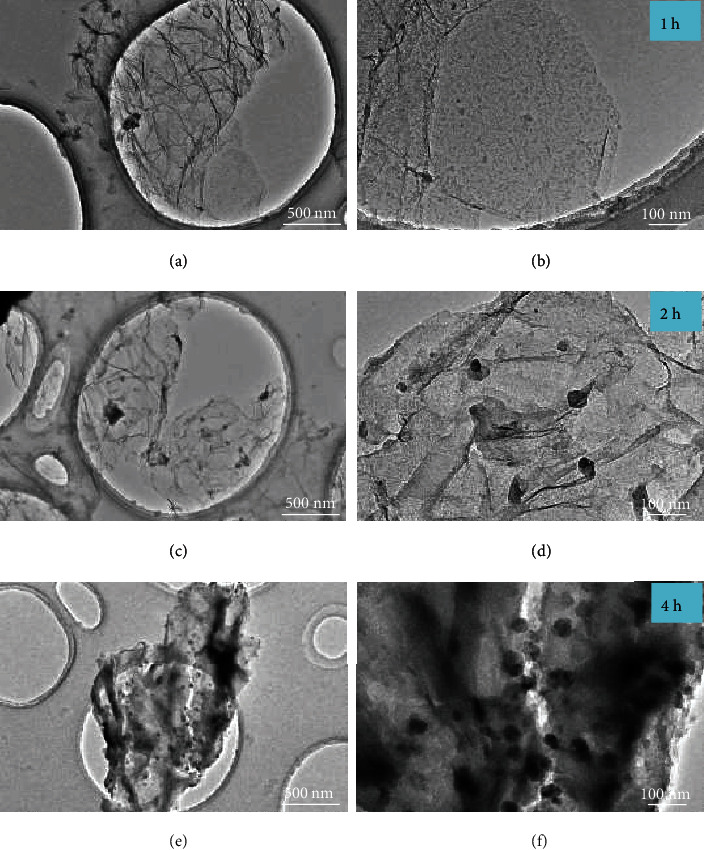
TEM images of NP-TiH_2_@G with the ultrasonic time of 1 h (a, b), 2 h (c, d), and 4 h (e, f).

A time dependence of growth of TiH_2_ nanoplates was also clearly observed by means of TEM (as shown in Figure 5). For comparison, the pristine graphene with a clean surface is shown in Figure S4 (Supporting Information). After 1 h of ultrasonic treatment, a large number of ~10 nm sized black sheets cover on the graphene (Figures 5(a) and 5(b)). Extending the time to 2 h, some nanoplates grew up to ~50 nm (Figures 5(c) and 5(d)). Further extending to 4 h, the ~50 nm-sized nanoplates were largely increased in quantity along with the disappearance of small nanosheets (Figures 5(e) and 5(f)). The loading amount of TiH_2_ was determined to be ~70% in weight by inductively coupled plasma spectroscopy (ICP) examination, which is distinctly higher than that obtained previously by a solvothermal process (~46%) [44]. Thus, higher catalytic activity was expected. In a strong contrast, only coarse particles with ~500 nm in size were obtained via the same sonochemical process without graphene as support (Figure S5, Supporting Information). This fact unambiguously indicates the critical important role played by graphene as a hard template governing the morphology of TiH_2_ nanoplate, attributed to the very similarity in lattice spacings (2.10 Å for the (002) planes of TiH_2_ and 2.13 Å for the (100) planes of graphene) [44].

### 2.2. Catalytic Activity of TiH_2_ Nanoplates

**Figure 6:**
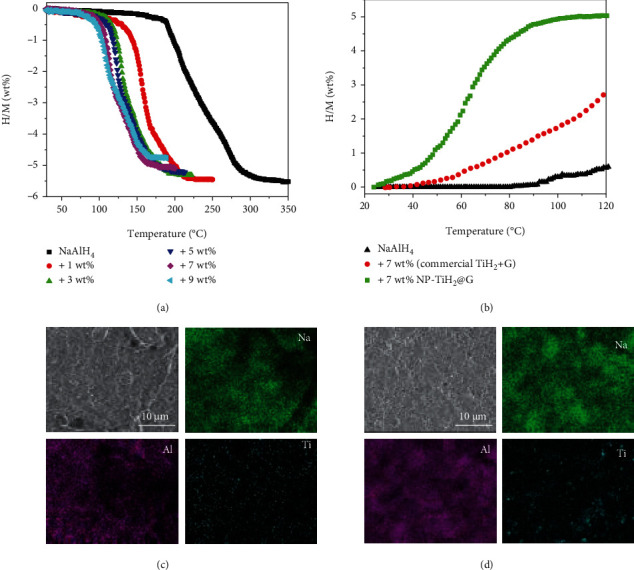
(a) Volumetric release curves of NaAlH_4_ doped with NP-TiH_2_@G, (b) nonisothermal hydrogenation curves, and (c, d) SEM and corresponding EDS mapping of NaAlH_4_ mixed with 7 wt% (c) TiH_2_ nanoplates and (d) commercial TiH_2_.

The 4 h-sonicated NP-TiH_2_@G was selected to mix with NaAlH_4_ by ball milling in order to evaluate its catalytic effectiveness because it took 4 h to complete the conversion of TiH_2_ from TiCl_4_ as indicated by the thorough disappearance of the characteristic reflections of LiH in the XRD profile after 4 h of sonification (Figure S6, Supporting Information). Six samples of NaAlH_4_-*x*NP-TiH_2_@G with *x* = 0, 1, 3, 5, 7, and 9 wt% were examined. A remarkable reduction in the dehydrogenation temperature of NaAlH_4_ was observed as NP-TiH_2_@G increasing from 1 wt% to 7 wt% (as shown in Figure 6(a)). For the 7 wt% NP-TiH_2_@G-containing sample, the release of hydrogen started from 80°C and completed at 160°C with an usable hydrogen capacity of 5 wt%. The on-set and end temperatures of dehydrogenation were reduced by 115 and 180°C, respectively, compared to those of the pristine NaAlH_4_. Further increase of NP-TiH_2_@G to 9 wt% caused an obvious loss of hydrogen capacity without obvious reduction in the dehydrogenation temperature. Therefore, 7 wt% was the optimal amount for NP-TiH_2_@G by taking into account of the hydrogen capacity and the dehydrogenation temperature.

The dehydrogenated sample was subsequently subjected to hydrogenation with ramped temperatures under 100 atm of H_2_ pressure (as shown in Figure 6(b)). The 7 wt% NP-TiH_2_@G-containg sample showed superior rehydrogenation properties to those of pristine NaAlH_4_ and NaAlH_4_ doped with the commercial TiH_2_. Specifically, 7 wt% NP-TiH_2_@G-containg sample absorbed 5 wt% H_2_ from 25°C to 105°C. It is worth emphasizing that the 7 wt% NP-TiH_2_@G-containing sample started to absorb hydrogen at a temperature as low as 25°C, and more than 90% of the rehydrogenation can be completed below 90°C, which is close to the working temperature of PEMFC. Such significant improvement of the rehydrogenation by the addition of 7 wt% NP-TiH_2_@G demonstrated for the first time that high reversible capacity coupled with the dehydrogenation temperature of NaAlH_4_ can be achieved simultaneously by a proper dopant, which can be mainly attributed to the highly homogenous dispersion of the prepared NP-TiH_2_@G (Figure 6(c)) than that of the commercial TiH_2_ (Figure 6(d)) in NaAlH_4_. The Ti-rich area was clearly observed in NaAlH_4_ doped with the commercial TiH_2_, probably due to the large particle size of TiH_2_ (Figure S7, Supporting Information). More importantly, most of the NP-TiH_2_ was converted to Al-Ti species after 24 h ball milling with NaAlH_4_, whereas only small amount of Al-Ti species can be detected in the commercial TiH_2_ (Figure S8, Supporting Information). This suggests that the reduced particle size of TiH_2_ facilitates the formation of Al-Ti species, which possess high catalytic activity for the dehydrogenation and rehydrogenation of NaAlH_4_ [9, 34, 40]. Further XRD characterization indicated that the reversible hydrogen capacity still originated from the decomposition and reformation of NaAlH_4_ (as shown in Figure S9 (Supporting Information)).

**Figure 7:**
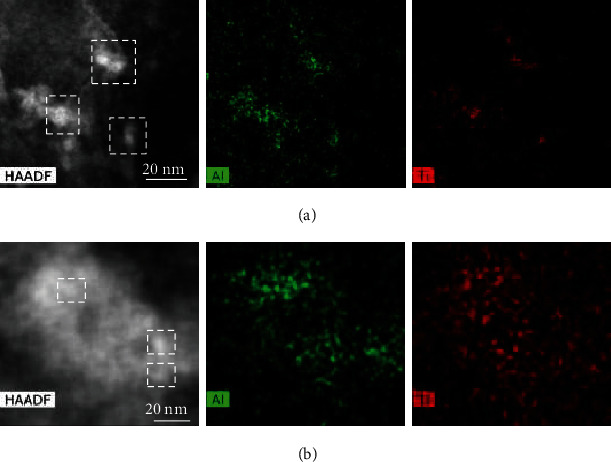
STEM and corresponding EDS mapping of as-milled (a) and activated (b) NaAlH_4_-7 wt% NP-TiH_2_@G samples. The rectangular areas in (a) and (b) are taken for composition analysis.

Moreover, the dehydrogenation temperature was further reduced by ~10°C in the follow-up 2nd cycle (Figure S10, Supporting Information). It can be clearly seen that the particle size of Ti-containing species reduced largely to around 5 nm from the aberration-corrected scanning transmission electron microscope (STEM) observation and EDS mapping analyses (as shown in Figure 7). From the relative content analyses, the Al-Ti species changed from Al_85_Ti_15_ for the as-milled sample to Al_58_Ti_42_ for the cycled sample, close to Al_50_Ti_50_, suggesting the reconstruction or optimization of the local atomic structure of Al-Ti species during cycling. This is further evidenced by the slight low-angle shift of the characteristic reflection of Al-Ti species in the XRD profiles because of the incorporation of more Ti (Figure S11, Supporting Information). According to the density functional theory (DFT) calculation, the kinetic barrier of the transfer of H atom from NaAlH_4_ to the surface of Al is largely reduced from 0.47 eV (Figure S12a, Supporting Information) to 0.14 eV (Figure S12b, Supporting Information) with the present of one Ti atom. This process can even proceed spontaneously when two Ti atoms are introduced into the surface of Al in the near-nearest-neighbor mode (Figure S12c, Supporting Information). This suggests that the Al-Ti species are of great importance for the significant improvement of dehydrogenation kinetics of NaAlH_4_, which agrees well with the previous reports [40, 45].

### 2.3. Hydrogen Storage Kinetics of NP-TiH_2_@G-Containing NaAlH_4_

**Figure 8:**
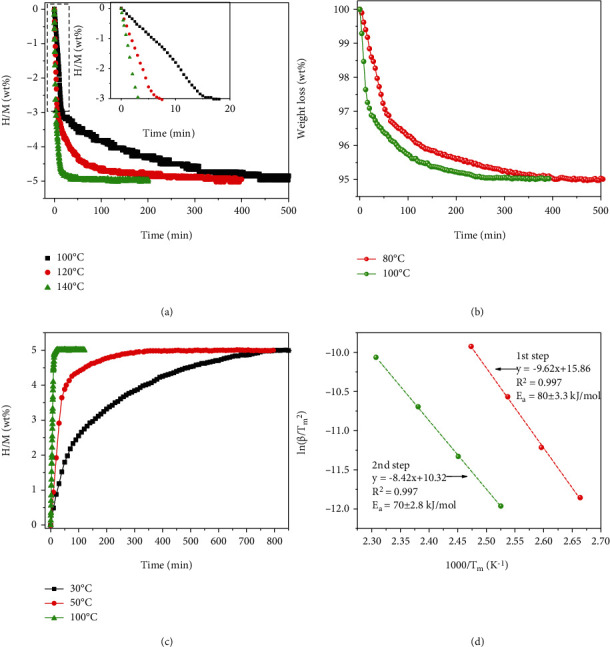
(a) Isothermal dehydrogenation curves, (b) isothermal TG curves, (c) isothermal hydrogenation curves, and (d) Kissinger's plots of activated NaAlH_4_-7 wt% NP-TiH_2_@G sample.

Figures 8(a) and 8(b) show the isothermal dehydrogenation behaviors of NaAlH_4_-7 wt% NP-TiH_2_@G sample after 1 dehydrogenation/rehydrogenation cycle, measured by volumetric and thermogravimetric (TG) methods, respectively. Isothermal volumetric dehydrogenation indicates that the full dehydrogenation of 5 wt% of hydrogen was achieved within 30 min at 140°C. At 120°C, it took around 200 min to complete. Even at 100°C, the major part of hydrogen (around 3.2 wt%) can be released within 30 min and the dehydrogenation completed within 500 min. The time for the full dehydrogenation was reduced to only 250 min at TG measurement (Figure 8(b)), which is attributed to the absence of blocking effect from hydrogen back pressure. More encouragingly, the full dehydrogenation can be achieved even at 80°C, which is the lowest dehydrogenation temperature for NaAlH_4_ reported so far.

The full isothermal rehydrogenation (~5 wt% of hydrogen) of dehydrogenated NaAlH_4_-7 wt% NP-TiH_2_@G completed within only 25 min at 100°C (as shown in Figure 8(c)). Amazingly, the full rehydrogenation was also achieved even at 30°C. This is the first complex hydride system that is able to work at the target temperature range proposed by US DOE with 5 wt% of reversible hydrogen capacity, although the dehydrogenation/rehydrogenation rates need to be further improved [46].

The apparent activation energy of dehydrogenation reactions of NaAlH_4_-7 wt% NP-TiH_2_@G was calculated based on the Kissinger's plots (Figure 8(d)), in which the peak temperatures and the heating rates were obtained from temperature programmed desorption (TPD) curves shown in Figure S13 (Supporting Information). The apparent activation energies for each step are 80 ± 3.3 and 70 ± 2.8 kJ mol^−1^, respectively. These values are reduced by ~40% compared to those of the pristine NaAlH_4_ [47], and even remarkably lower than those of other catalyst-modified NaAlH_4_ systems (Table S1) [48–54], indicating the significant reduction of the dehydrogenation kinetic barriers induced by the newly formed Al-Ti catalytic species. In contrast, the addition of 7 wt% NP-TiH_2_@G did not affect much the thermodynamic properties of NaAlH_4_ as indicated by the nearly unchanged desorption enthalpy change, which were determined to be approximately 36.5/47.4 and 36.3/47.0 kJ/mol-H_2_ for pristine sample and 7 wt% NP-TiH_2_@G-containing sample, respectively, by analyzing the differential scanning calorimetry (DSC) results (Figure S14, Supporting Information).

### 2.4. Dehydrogenation/Rehydrogenation Cycling of NP-TiH_2_@G-Containing NaAlH_4_

**Figure 9:**
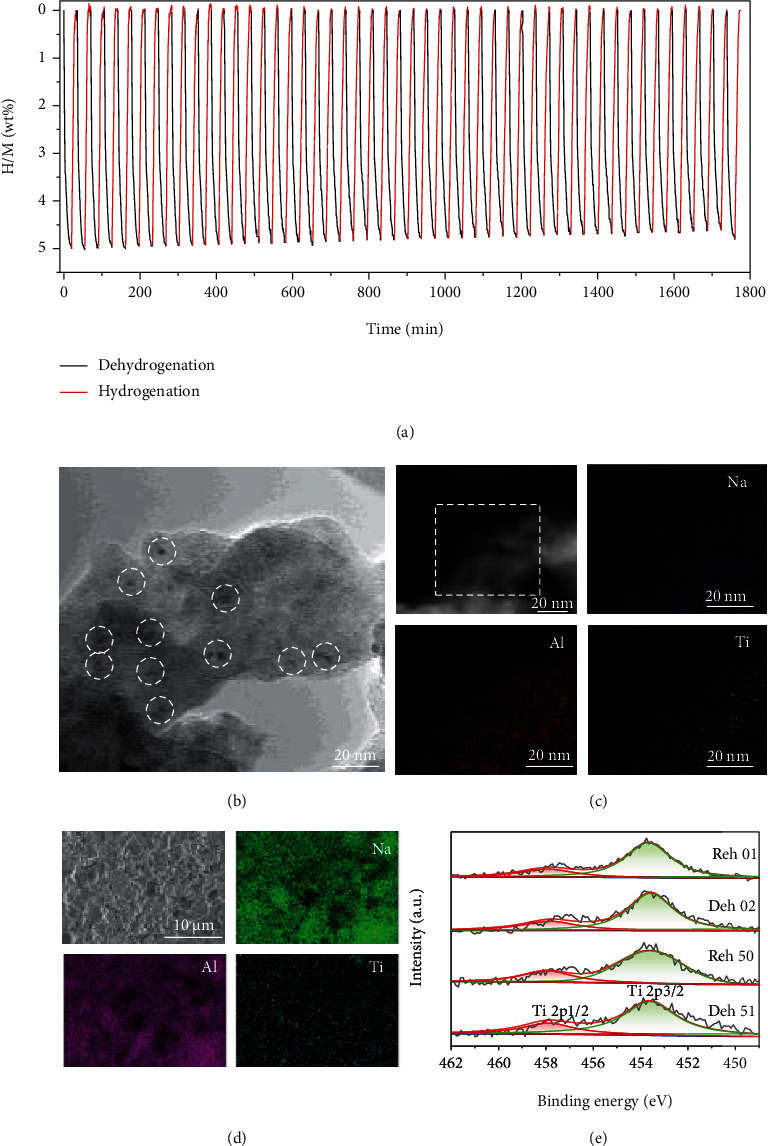
(a) Cycling tests operated at 140°C for dehydrogenation and 100°C/100 atm H_2_ for hydrogenation of NaAlH_4_-7 wt% NP-TiH_2_@G, (b) TEM image, (c) STEM and corresponding EDS mapping images, (d) SEM and corresponding images, and (e) Ti 2p XPS spectra of NaAlH_4_-7 wt% NP-TiH_2_@G sample after 50 cycles.

Dehydrogenation/rehydrogenation cycling performance of the NaAlH_4_-7 wt% NP-TiH_2_@G sample is shown in Figure 9(a). Here, dehydrogenation was operated at 140°C under initial vacuum and rehydrogenation at 100°C/100 atm H_2_. No obvious degradation was observed after 50 cycles. The hydrogen capacity was 4.8 wt% at the 50th cycle, which means a capacity retention of 96% based on the initial capacity of 5.0 wt%. The cycling performance demonstrates a stable cyclability of the NP-TiH_2_@G-containing NaAlH_4_.

To shed light on the stable cycling behavior of NP-TiH_2_@G-containing NaAlH_4_, the particle size, distribution, and chemical states of catalytic species were examined and analyzed. TEM observation displayed that the catalytic species remained as ultrafine particles of ~5 nm in size without obvious agglomeration (Figure 9(b)). EDS mapping analyses (Figures 9(c) and 9(d)) indicated the homogenous distribution of Ti element on NaAlH_4_ matrix even after 50 cycles. In addition, high-resolution XPS spectra of Ti 2p showed a stable chemical state from 2 to 50 cycles for the nearly unchanged 2p_3/2_-2p_1/2_ spin–orbit doublet at 453.2/458.1 eV (Figure 9(e)) [55]. As a result, we believe that the small particle size, homogenous dispersion, and stable chemical state are of critical importance for the long-term cyclability of NP-TiH_2_@G-containing NaAlH_4_. This finding provides important insights and greatly encourages the further development of the catalysis-promoted complex hydrides for practical on-board applications.

## 3. Discussion

Two-dimensional TiH_2_ nanoplates with a lateral size of 50 nm and a thickness of 15 nm were successfully synthesized by using graphene as support, based on a novel facile sonochemical process. The graphene played a critical role in the nucleation and growth of TiH_2_ nanoplates. The prepared TiH_2_ nanoplates displayed superior catalytic activity than the commercial TiH_2_ of microscale for hydrogen storage in NaAlH_4_. The 7 wt% NP-TiH_2_@G-containing NaAlH_4_ started releasing hydrogen at 80°C, which was lowered by 115°C in comparison with pristine sample. In TG measurement, full dehydrogenation was achieved with 5.0 wt% of practical hydrogen capacity even at 80°C. It is worth emphasizing that the rehydrogenation can complete at 30°C under 100 atm of H_2_. Operating at 140°C/initial vacuum for dehydrogenation and 100°C/100 atm H_2_ for rehydrogenation, a stable cyclability was confirmed, as only 0.2 wt% of capacity loss after 50 cycles. Mechanistic studies revealed the active catalytic species was converted from TiH_2_ to Al_85_Ti_15_ during ball milling and further to near Al_50_Ti_50_ after the first de-/hydrogenation cycle, which remained stable in the subsequent cycling. DFT calculations reveal that the kinetic barrier of the transfer of H atom from NaAlH_4_ to the surface of Al is largely reduced by the formation of Al-Ti species. The small particle, homogenous dispersion and stable chemical state of active catalytic species are responsible for the long-term cyclability of NP-TiH_2_@G-containing NaAlH_4_. The findings presented in this work make NaAlH_4_ step closer towards practical on-board hydrogen storage applications.

## 4. Materials and Methods

### 4.1. Materials Synthesis

All reagents and solvents were purchased and used as received without further purification. TiH_2_ nanoplates supported on graphene (NP-TiH_2_@G) were synthesized by a newly developed sonochemical process [56] under argon atmosphere using titanium chloride (TiCl_4_, 99.9%, Aladdin), lithium hydride (LiH, 99.4%, Alfa Aesar), and graphene (97%, Aladdin) as the raw materials. In a typical procedure, TiCl_4_ (2 mmol), LiH (8 mmol), and graphene (20 mg) were sequentially added to 70 mL THF in a flask-3-neck which was irradiated by ultrasonic waves (40 kHz, W-600D, Shanghai Ultrasonic Instrument, Shanghai, China) for 4 h under mechanical stirring. A black precipitate of NP-TiH_2_@G was separated from the THF solution by filtration, washed twice with THF and finally dried at 70°C under dynamic vacuum. The obtained NP-TiH_2_@G was mixed with NaAlH_4_ on a planetary ball mill (Nanjing, China). The ball milling was conducted at 500 rpm for 24 h in the milling jar filled with 50 atm H_2_ at the ball-to-sample weight ratio of approximately 120 : 1. The doping amounts of NP-TiH_2_@G were set to be *x* = 0, 1, 3, 5, 7, and 9 wt%.

### 4.2. Characterization

The structure information was collected on a MiniFlex 600 X-ray diffractometer (XRD) (Rigaku, Japan) with Cu K_*α*_ radiation (*λ* = 0.15406 nm) operated at 40 kV and 15 mA. The 2*θ* range was set at 10-90° with a 0.05° step increment. A custom-designed container with a window covered by Scotch tape was used to prevent air and moisture exposure of the sample. The sample morphology and microstructure were observed with scanning electron microscope (SEM) (Hitachi S-4800), aberration-corrected scanning transmission electron microscope (STEM) (Titan G^2^ 80-200 Chemi STEM FEI, 200 kV), and TEM (Tecnai G^2^ F20 S-TWIN FEI, 200 kV). For SEM observation, the sample was transferred quickly to the SEM facility under Ar protection. For STEM and TEM examinations, the sample was protected with a double-tilt vacuum transfer holder (Gatan 648, USA). Atomic force microscope (AFM) characterization was performed on Bruker Dimension Icon under the tapping mode, with samples prepared by dropping freshly diluted sample solutions onto silicon substrates. X-ray photoelectron spectroscopy (XPS) analyses were carried out using a Thermo Scientific ESCALAB 250Xi spectrometer with a monochromatic Al Ka X-ray source at a base pressure of 6.8 × 10^−9^ Torr. The Ti content of samples were determined by inductively coupled plasma (ICP) spectroscopy on a PE Optima 8000 instrument.

### 4.3. Property Measurements

A home-built temperature programmed desorption (TPD) system attached to a mass spectrometer (MS) was employed to characterize the temperature-dependent dehydrogenation behavior using Ar as a carrier gas with a flow rate of 20 mL min^−1^. For each test, approximately 40 mg sample was heated up from room temperature to desired temperatures at 2°C min^−1^. Quantitative dehydrogenation/hydrogenation properties were measured using a Sieverts-type apparatus under isothermal and nonisothermal conditions, and the sample loading was approximately 70 mg sample. The nonisothermal data were acquired by gradually heating the sample from room temperature to a preset temperature at an average rate of 2°C min^−1^ under primary vacuum (-10^−3^ Torr) for dehydrogenation and 1°C min^−1^ with 100 atm H_2_ for hydrogenation. The isothermal measurements were conducted by rapidly heating the sample to a desired temperature and then dwelling during the entire test. The temperature and pressure were monitored and recorded simultaneously, and the amounts of hydrogen released/uptaken were calculated based upon the ideal gas law. Thermogravimetric analysis (TGA) was carried out on a Netzsch TG 209 F3 instrument under an argon atmosphere at a ramping rate of 2°C min^−1^. Differential scanning calorimetry (DSC) experiments were performed with a NETZSCH DSC 200F3 unit at 2°C min^−1^ of heating rate. Approximately 2 mg of sample was placed in an Al_2_O_3_ crucible for measurement.

### 4.4. Theoretical Calculation

Density functional theory (DFT) calculations were conducted in the Vienna Ab initio Simulation Package (VASP). The generalized gradient approximation (GGA) with Perdew-Burke-Ernzerhof (PBE) model was taken as the exchange-correlation functional [57]. Projector augmented wave pseudopotentials (PAWs) were employed to model the ionic potentials [58]. The precession setting of “PREC = Accurate” was used. All atoms were fully relaxed until the force on them was less than 0.05 eV Å^−1^. The Brillouin zone integration was performed with gamma-centred sampling of 3 × 3 × 1. The minimum-energy pathway was computed using the climbing-image nudged elastic band (CI-NEB) method [59]. Al(111) surface was selected because it has the lowest surface free energy and then is most likely exposed. A six-layer slab containing 96 atoms was constructed to simulate the surface with the lowest two layers fixed to represent the bulk. The thickness of vacuum layer was set as 20 Å to avoid interaction between neighbouring images.

## Data Availability

The data used to support the findings of this study are included within the article and supplementary information files and/or may be requested from the authors.

## Supplementary Materials

Supplementary Materials Figure S1: Raman spectrum of as-prepared solid product after sonochemical reaction between TiCl_4_ and LiH in THF. Figure S2: MS signal of gaseous product of reaction of TiCl_4_ with LiH in THF under ultrasonic treatment. Figure S3: XRD profile of the solid obtained by drying the filtrate of reaction of TiCl_4_ with LiH in THF under ultrasonic treatment. Figure S4: TEM image of pristine graphene. Figure S5: SEM image of TiH_2_ prepared by sonochemical reaction of TiCl_4_ and LiH without graphene as support. Figure S6: XRD profiles of NaAlH_4_ doped by 7 wt% NF-TiH_2_@G prepared by different ultrasonic times. Figure S7: SEM image of commercial TiH_2_. Figure S8: Ti 2p XPS spectra of NaAlH_4_ mixed with commercial TiH_2_ and TiH_2_ nanoplates. Figure S9: XRD profiles of 7 wt% NF-TiH_2_@G-containing NaAlH_4_ after different treatments. Figure S10: the volumetric dehydrogenation curves of activated NaAlH4-7 wt% NP-TiH2@G sample. Figure S11: XRD profiles of NaAlH_4_-7 wt% NP-TiH_2_@G sample after ball milling and 1st de/rehydrogenation cycle (2*θ*: 39-44°). Figure S12: energy barriers of H atom transferring from NaAlH_4_ molecule to Al surface (a) and single-Ti-substituted Al surface (b) and relaxed geometry of NaAlH_4_ molecule placed on two-Ti-substituted Al surface (c). Figure S13: TPD curves of activated NaAlH_4_-7 wt% NP-TiH_2_@G sample with different heating rates. Figure S14: DSC curves of pristine NaAlH_4_ and NaAlH_4_-7 wt% NP-TiH_2_@G samples. Table S1: comparison of activation energy (Ea) of NaAlH_4_ doped with different catalysts.

## References

[1] Schlapbach L., Züttel A. (2001). Hydrogen-storage materials for mobile applications. *Nature*.

[2] Zheng J., Wang C.-G., Zhou H. (2021). Current research trends and perspectives on solid-state nanomaterials in hydrogen storage. *Research*.

[3] Lang C. G., Jia Y., Yao X. (2020). Recent advances in liquid-phase chemical hydrogen storage. *Energy Storage Materials*.

[4] He T., Pachfule P., Wu H., Xu Q., Chen P. (2016). Hydrogen carriers. *Nature Reviews Materials*.

[5] Orimo S.-I., Nakamori Y., Eliseo J. R., Züttel A., Jensen C. M. (2007). Complex hydrides for hydrogen storage. *Chemical Reviews*.

[6] Ouyang L. Z., Chen K., Jiang J., Yang X. S., Zhu M. (2020). Hydrogen storage in light-metal based systems: a review. *Journal of Alloys and Compounds*.

[7] Li L., Huang Y., An C., Wang Y. (2019). Lightweight hydrides nanocomposites for hydrogen storage: challenges, progress and prospects. *Science China Materials*.

[8] Yu X. B., Tang Z., Sun D., Ouyang L., Zhu M. (2017). Recent advances and remaining challenges of nanostructured materials for hydrogen storage applications. *Progress in Materials Science*.

[9] Frankcombe T. J. (2012). Proposed mechanisms for the catalytic activity of Ti in NaAlH_4_. *Chemical Reviews*.

[10] Bogdanović B., Felderhoff M., Pommerin A., Schüth F., Spielkamp N. (2006). Advanced hydrogen-storage materials based on Sc-, Ce-, and Pr-doped NaAlH_4_. *Advanced Materials*.

[11] Ali N. A., Ismail M. (2021). Modification of NaAlH_4_ properties using catalysts for solid-state hydrogen storage: a review. *International Journal of Hydrogen Energy*.

[12] Liu Y. F., Ren Z. H., Zhang X. (2018). Development of catalyst-enhanced sodium alanate as an advanced hydrogen-storage material for mobile applications. *Energy Technology*.

[13] Zhang X. L., Liu Y. F., Zhang X., Hu J. J., Gao M. X., Pan H. G. (2020). Empowering hydrogen storage performance of MgH_2_ by nanoengineering and nanocatalysis. *Materials Today Nano*.

[14] Zhang W. X., Zhang X., Huang Z. G. (2021). Recent development of lithium borohydride-based materials for hydrogen storage. *Advanced Energy and Sustainability Research*.

[15] Bogdanović B., Schwickardi M. (1997). Ti-doped alkali metal aluminium hydrides as potential novel reversible hydrogen storage materials^1^. *Journal of Alloys and Compounds*.

[16] Lee G., Shim J., Cho Y., Lee K. (2008). Improvement in desorption kinetics of NaAlH_4_ catalyzed with TiO_2_ nanopowder. *International Journal of Hydrogen Energy*.

[17] Eigen N., Kunowsky M., Klassen T., Bormann R. (2007). Synthesis of NaAlH_4_-based hydrogen storage material using milling under low pressure hydrogen atmosphere. *Journal of Alloys and Compounds*.

[18] Xiao X. Z., Yu K. R., Fan X. L. (2011). Synthesis and hydriding/dehydriding properties of nanosized sodium alanates prepared by reactive ball-milling. *International Journal of Hydrogen Energy*.

[19] Zou G. D., Liu B. Z., Guo J. X., Zhang Q., Fernandez C., Peng Q. (2017). Synthesis of nanoflower-shaped Mxene derivative with unexpected catalytic activity for dehydrogenation of sodium alanates. *ACS Applied Materials & Interfaces*.

[20] Xiao X. Z., Fan X. L., Yu K. R. (2009). Catalytic mechanism of new TiC-doped sodium alanate for hydrogen storage. *Journal of Physical Chemistry C*.

[21] Yuan Z. L., Zhang D. F., Fan G. X., Chen Y., Fan Y., Liu B. (2021). Synergistic effect of CeF_3_ Nanoparticles supported on Ti_3_C_2_ MXene for catalyzing hydrogen storage of NaAlH_4_. *ACS Applied Energy Materials*.

[22] Jiang R. C., Xiao X., Zheng J., Chen M., Chen L. (2020). Remarkable hydrogen absorption/desorption behaviors and mechanism of sodium alanates in-situ doped with Ti-based 2D Mxene. *Materials Chemistry and Physics*.

[23] Kim J. W., Shim J.-H., Kim S. C. (2009). Catalytic effect of titanium nitride nanopowder on hydrogen desorption properties of NaAlH_4_ and its stability in NaAlH_4_. *Journal of Power Sources*.

[24] Li L., Qiu F., Wang Y. (2012). Tin catalyst for the reversible hydrogen storage performance of sodium alanate system. *Journal of Materials Chemistry*.

[25] Li L., Qiu F. Y., Wang Y. J. (2012). Crystalline TiB_2_: an efficient catalyst for synthesis and hydrogen desorption/absorption performances of NaAlH_4_ system. *Journal of Materials Chemistry*.

[26] Xiao X., Chen L., Wang X., Li S., Chen C., Wang Q. (2008). Reversible hydrogen storage properties and favorable co-doping mechanism of the metallic Ti and Zr co-doped sodium aluminum hydride. *International Journal of Hydrogen Energy*.

[27] Wang P., Jensen C. M. (2004). Preparation of Ti-doped sodium aluminum hydride from mechanical milling of NaH/Al with off-the-shelf Ti powder. *Journal of Physical Chemistry B*.

[28] Pitt M. P., Vullum P. E., Sørby M. H. (2012). Hydrogen absorption kinetics and structural features of NaAlH_4_ enhanced with transition-metal and Ti-based nanoparticles. *International Journal of Hydrogen Energy*.

[29] Zhang X., Zhang X. L., Ren Z. H. (2020). Amorphous-carbon-supported ultrasmall TiB_2_ nanoparticles with high catalytic activity for reversible hydrogen storage in NaAlH_4_. *Frontiers in Chemistry*.

[30] Zhang X., Ren Z. H., Lu Y. H. (2018). Facile synthesis and superior catalytic activity of nano-TiN@N-C for hydrogen storage in NaAlH_4_. *ACS Applied Materials & Interfaces*.

[31] Wu R. Y., du H., Wang Z. Y., Gao M., Pan H., Liu Y. (2016). Remarkably improved hydrogen storage properties of NaAlH_4_ doped with 2D titanium carbide. *Journal of Power Sources*.

[32] Schüth F., Bogdanović B., Felderhoff M. (2004). Light metal hydrides and complex hydrides for hydrogen storage. *Chemical Communications*.

[33] Zhang S., Lu C., Takeichi N., Kiyobayashi T., Kuriyama N. (2011). Reaction stoichiometry between TiCl_3_ and NaAlH_4_ in Ti-doped alanate for hydrogen storage: the fate of the titanium species. *International Journal of Hydrogen Energy*.

[34] Léon A., Schild D., Fichtner M. (2005). Chemical state of Ti in sodium alanate doped with TiCl_3_ using X-ray photoelectron spectroscopy. *Journal of Alloys and Compounds*.

[35] Wang P., Kang X. D., Cheng H. M. (2005). Improved hydrogen storage of TiF3-Doped NaAlH_4_. *ChemPhysChem*.

[36] Li L., Qiu F. Y., Wang Y. J. (2012). Improved dehydrogenation performances of TiB_2_-doped sodium alanate. *Materials Chemistry and Physics*.

[37] Wang T., Wang J., Ebner A. D., Ritter J. A. (2008). Reversible hydrogen storage properties of NaAlH_4_ catalyzed with scandium. *Journal of Alloys and Compounds*.

[38] Gross K. J., Majzoub E. H., Spangler S. W. (2003). The effects of titanium precursors on hydriding properties of alanates. *Journal of Alloys and Compounds*.

[39] Kang X., Wang P., Cheng H. (2007). In situ formation of Ti hydride and its catalytic effect in doped NaAlH_4_ prepared by milling NaH/Al with metallic Ti powder. *International Journal of Hydrogen Energy*.

[40] Zhang X., Liu Y., Wang K., Gao M., Pan H. (2015). Remarkably improved hydrogen storage properties of nanocrystalline TiO_2_-modified NaAlH_4_ and evolution of Ti-containing species during dehydrogenation/hydrogenation. *Nano Research*.

[41] Dathara G. K. P., Mainardi D. S. (2008). Structure and dynamics of Ti–Al–H compounds in Ti-doped NaAlH_4_. *Molecular Simulation*.

[42] Dathar G. K. P., Mainardi D. S. (2010). Kinetics of hydrogen desorption in NaAlH_4_ and Ti-containing NaAlH_4_. *Journal of Physical Chemistry C*.

[43] Íñiguez J., Yildirim T. (2005). First-principles study of Ti-doped sodium alanate surfaces. *Applied Physics Letters*.

[44] Ren Z. H., Zhang X., Huang Z. G. (2022). Controllable synthesis of 2D TiH_2_ nanoflakes with superior catalytic activity for low-temperature hydrogen cycling of NaAlH_4_. *Chemical Engineering Journal*.

[45] Zhang X., Ren Z. H., Zhang X. L., Gao M., Pan H., Liu Y. (2019). Triggering highly stable catalytic activity of metallic titanium for hydrogen storage in NaAlH_4_ by preparing ultrafine nanoparticles. *Journal of Materials Chemistry A*.

[46] US Department of Energy (2016). DOE Technical Targets for Onboard Hydrogen Storage for Light-Duty Vehicles. https://www.energy.gov/eere/fuelcells/doe-technical-targets-onboard-hydrogen-storage-light-duty-vehicles.

[47] Zhang X., Wu R. Y., Wang Z. Y., Gao M., Pan H., Liu Y. (2016). Preparation and catalytic activity of a novel nanocrystalline ZrO_2_@C composite for hydrogen storage in NaAlH_4_. *Chemistry-An Asian Journal*.

[48] Liu Y. F., Liang C., Zhou H., Gao M., Pan H., Wang Q. (2011). A novel catalyst precursor K_2_TiF_6_ with remarkable synergetic effects of K, Ti and F together on reversible hydrogen storage of NaAlH_4_. *Chemical Communications*.

[49] Li L., Wang Y., Qiu F. Y. (2013). Reversible hydrogen storage properties of NaAlH_4_ enhanced with TiN catalyst. *Journal of Alloys and Compounds*.

[50] Idris N. H., Anuar A. S. K., Ali N. A., Ismail M. (2021). Effect of K_2_NbF_7_ on the hydrogen release behaviour of NaAlH_4_. *Journal of Alloys and Compounds*.

[51] Mao J. F., Guo Z., Liu H. (2011). Improved hydrogen sorption performance of NbF_5_-catalysed NaAlH_4_. *International Journal of Hydrogen Energy*.

[52] Mustafa N. S., Yahya M. S., Sazelee N., Ali N. A., Ismail M. (2018). Dehydrogenation properties and catalytic mechanism of the K_2_NiF_6_-doped NaAlH_4_ System. *ACS Omega*.

[53] Sazelee N., Mustafa N. S., Yahya M. S., Ismail M. (2021). Enhanced dehydrogenation performance of NaAlH_4_ by the addition of spherical SrTiO3. *International Journal of Energy Research*.

[54] Fan X. L., Xiao X. Z., Chen L. X. (2011). Hydriding-dehydriding kinetics and the microstructure of La- and Sm-doped NaAlH_4_ prepared via direct synthesis method. *International Journal of Hydrogen Energy*.

[55] Mencer D. E., Hess T. R., Mebrahtu T., Cocke D. L., Naugle D. G. (1991). Surface reactivity of titanium–aluminum alloys: Ti_3_Al, TiAl, and TiAl_3_. *Journal of Vacuum Science & Technology A: Vacuum, Surfaces, and Films*.

[56] Zhang X., Liu Y. F., Ren Z. H. (2021). Realizing 6.7 wt% reversible storage of hydrogen at ambient temperature with non-confined ultrafine magnesium hydrides. *Energy & Environmental Science*.

[57] Perdew J. P., Burke K., Ernzerhof M. (1996). Generalized gradient approximation made simple. *Physical Review Letters*.

[58] Blöchl P. E. (1994). Projector augmented-wave method. *Physical Review B*.

[59] Henkelman G., Uberuaga B. P., Jónsson H. (2000). A climbing image nudged elastic band method for finding saddle points and minimum energy paths. *Journal of Chemical Physics*.

