# Supplementary material for: Titanium Hydride Nanoplates Enable 5 wt% of Reversible Hydrogen Storage by Sodium Alanate below 80°C
Source: Research (Wash D C). 2021 Dec 14;2021:9819176. doi: 10.34133/2021/9819176 (PMC8696284; doi:10.34133/2021/9819176)
Supplement: Supplementary Materials — Figure S1: Raman spectrum of as-prepared solid product after sonochemical reaction between TiCl4 and LiH in THF. Figure S2: MS signal of gaseous product of reaction of TiCl4 with LiH in THF under ultrasonic treatment. Figure S3: XRD profile of the solid obtained by drying the filtrate of reaction of TiCl4 with LiH in THF under ultrasonic treatment. Figure S4: TEM image of pristine graphene. Figure S5: SEM image of TiH2 prepared by sonochemical reaction of TiCl4 and LiH without graphene as support. Figure S6: XRD profiles of NaAlH4 doped by 7 wt% NF-TiH2@G prepared by different ultrasonic times. Figure S7: SEM image of commercial TiH2. Figure S8: Ti 2p XPS spectra of NaAlH4 mixed with commercial TiH2 and TiH2 nanoplates. Figure S9: XRD profiles of 7 wt% NF-TiH2@G-containing NaAlH4 after different treatments. Figure S10: the volumetric dehydrogenation curves of activated NaAlH4-7 wt% NP-TiH2@G sample. Figure S11: XRD profiles of NaAlH4-7 wt% NP-TiH2@G sample after ball milling and 1st de/rehydrogenation cycle (2θ: 39-44°). Figure S12: energy barriers of H atom transferring from NaAlH4 molecule to Al surface (a) and single-Ti-substituted Al surface (b) and relaxed geometry of NaAlH4 molecule placed on two-Ti-substituted Al surface (c). Figure S13: TPD curves of activated NaAlH4-7 wt% NP-TiH2@G sample with different heating rates. Figure S14: DSC curves of pristine NaAlH4 and NaAlH4-7 wt% NP-TiH2@G samples. Table S1: comparison of activation energy (Ea) of NaAlH4 doped with different catalysts. [file 9819176.f1.docx]

**Supplementary Materials**

Titanium hydride nanoplates enable 5 wt% of reversible hydrogen storage by sodium alanate below 80 °C

Zhuanghe Ren,^1†^ Xin Zhang,^1†^ Hai-Wen Li,^2^ Zhenguo Huang,^3^ Jianjiang Hu,*^,4^ Mingxia Gao,^1^ Hongge Pan,*^,1,5^ Yongfeng Liu*^,1,5^

^1^State Key Laboratory of Silicon Materials and School of Materials Science and Engineering, Zhejiang University, Hangzhou 310027, China.

^2^Hefei General Machinery Research Institute, Hefei 230031, China.

^3^School of Civil & Environmental Engineering, University of Technology Sydney, 81 Broadway, Ultimo, NSW, 2007, Australia.

^4^School of Chemistry and Chemical Engineering, Yantai University, Yantai 264005, China.

^5^Institute of Science and Technology for New Energy, Xi’an Technological University, Xi’an, 710021, China.

^†^These authors contributed equally to this work.

*Corresponding author. Email: [mselyf@zju.edu.cn (Y.F.L.)](mailto:mselyf@zju.edu.cn%20(Y.F.L.)), [jjj_hu@163.com](mailto:jjj_hu@163.com) (J.J.H.), [hgpan@zju.edu.cn](mailto:hgpan@zju.edu.cn) (H.G.P.)

**Figure S1.** Raman spectrum of as-prepared solid product after sonochemical reaction between TiCl_4_ and LiH in THF.

**Figure S2.** H_2_ signal of TPD-MS of commercial micro-sized TIH_2_

**Figure S3.** MS signal of gaseous product of reaction of TiCl_4_ with LiH in THF under ultrasonic treatment.

**Figure S4.** XRD profile of the solid obtained by drying the filtrate of reaction of TiCl_4_ with LiH in THF under ultrasonic treatment.

**Figure S5.** TEM image of pristine graphene.

**Figure S6.** SEM image of TiH_2_ prepared by sonochemical reaction of TiCl_4_ and LiH without graphene as support.

**Figure S7.** XRD profiles of NaAlH_4_ doped by 7 wt% NF-TiH_2_@G prepared by different ultrasonic times.

**Figure S8.** SEM image of commercial TiH_2_.

**Figure S9.** Ti 2p XPS spectra of NaAlH_4_ mixed with commercial TiH_2_ and TiH_2_ nanoplates.

**Figure S10.** XRD profiles of 7 wt% NF-TiH_2_@G-containing NaAlH_4_ after different treatments.

**Figure S11.** The volumetric dehydrogenation curves of activated NaAlH_4_-7 wt% NP-TiH_2_@G sample.

**Figure S12.** XRD profiles of NaAlH_4_-7 wt% NP-TiH_2_@G sample after ball milling and 1st de/rehydrogenation cycle (2θ: 39-44°).

**Figure S13.** Energy barriers of H atom transferring from NaAlH_4_ molecule to Al surface (a) and single-Ti-substituted Al surface (b) and relaxed geometry of NaAlH_4_ molecule placed on two-Ti-substituted Al surface (c).

**Figure S14.** TPD curves of activated NaAlH_4_-7 wt% NP-TiH_2_@G sample with different heating rates.

**Figure S15.** DSC curves of pristine NaAlH_4_ and NaAlH_4_-7 wt% NP-TiH_2_@G samples.

**Table S1**. Comparison of activation energy (E_a_) of NaAlH_4_ doped with different catalysts.

| Catalysts | 1st-step E_a_ (kJ/mol) | 2nd-step E_a_ (kJ/mol) | References |
| --- | --- | --- | --- |
| K_2_TiF_6_ | 99.8 | 88.0 | 48 |
| TiN | 91.7 | 99.9 | 49 |
| K_2_NbF_7_ | 84.6 | 75.1 | 50 |
| NbF_5_ | 88.2 | 102.9 | 51 |
| K_2_NiF_6_ | 89.9 | 99.6 | 52 |
| SrTiO_3_ | 79 | 92 | 53 |
| LaCl_3_ | 86.4 | 96.1 | 54 |
| La_3_Al_11_ | 93.0 | 99.3 | 54 |
| SmCl_3_ | 89.0 | 96.8 | 54 |
| SmAl_3_ | 91.9 | 98.9 | 54 |
| TiH_2_ nanoplates | 80 | 70 | This work |
